# Mass-produced and uniformly luminescent photochromic fibers toward future interactive wearable displays

**DOI:** 10.1038/s41377-024-01414-4

**Published:** 2024-04-02

**Authors:** Yan Li, Yikai Su

**Affiliations:** Department of Electronic Engineering, Shanghai Jiao Tong University, 200240 Shanghai, China

**Keywords:** Displays, Fibre lasers

## Abstract

Enabling flexible fibers with light-emitting capabilities has the potential to revolutionize the design of smart wearable interactive devices. A recent publication in Light Science & Application, an interdisciplinary team of scientists led by Prof. Yan-Qing Lu and Prof. Guangming Tao has realized a highly flexible, uniformly luminescent photochromic fiber based on a mass-produced thermal drawing method. It overcomes the shortcomings of existing commercial light-diffusing fibers, exhibiting outstanding one-dimensional linear illumination performance. The research team integrated controllable photochromic fibers into various wearable interaction interfaces, providing a novel approach and insights to enable human-computer interaction.

Wearable displays, including head-mounted displays^1,2^, smartwatches, and other innovative devices, have emerged as a new human-machine interface to realize the seamless interactions between humans and machines, offering advantages that traditional displays could not provide. Light-emitting fibers^3–8^, as a promising candidate for flexible wearable displays, can be woven, knitted, or embroidered into fabrics, providing more natural and versatile interactions (Fig. 1a). However, significant challenges remain in developing fiber devices when it comes to achieving uniform and customizable light effects while utilizing lightweight hardware. The current commercial light-emitting fiber is Corning® Fibrance® light-diffusing fibers^9^. The total internal reflection condition, which governs light propagation inside the fiber, was deliberately broken by introducing structural or material defects, thus actively inducing light leakage from the fiber. However, due to transmission losses and artificial defects, the brightness uniformity of the fiber in the transmission direction and the leakage uniformity in the circumferential direction cannot be guaranteed. Other light-emitting fibers based on optical waveguides are limited by their structural design or preparation processes^10–12^. Their length may not meet application requirements, and they may be susceptible to external interference that affects their light-emitting properties. In addition, fiber-based luminescent devices are often restricted by manufacturing processes and functional materials, as a single fiber can only emit a specific wavelength in spectral radiation^13^.

**Fig. 1:**
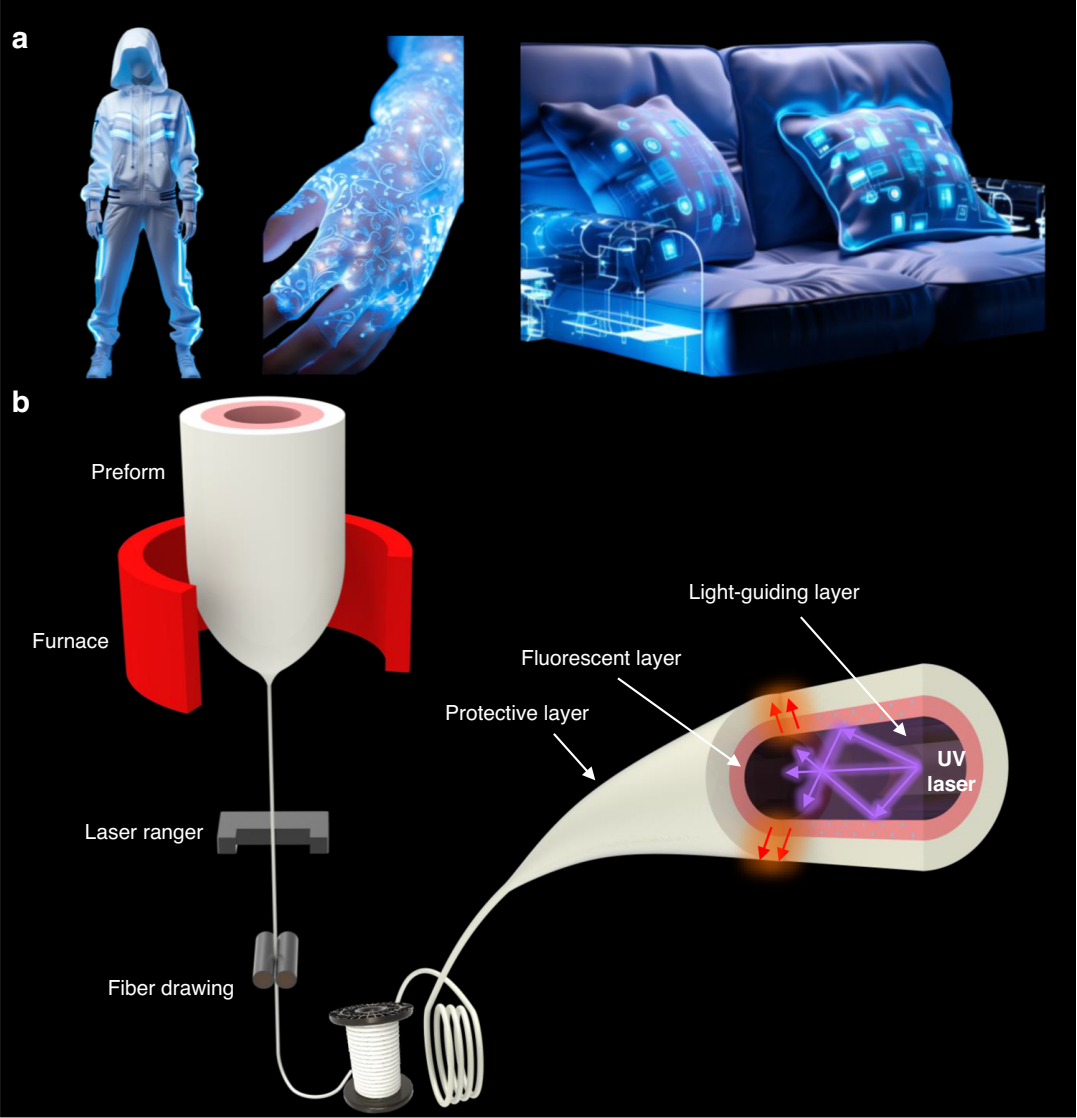
Wearable textile displays based on light-emitting fibers. **a** Application scenarios. **b** Fabrication and structure of the photochromic fiber

In a recent publication in *Light Science & Application*^14^, a team of scientists led by Professor Yan-Qing Lu of Nanjing University, and Professor Guangming Tao from Huazhong University of Science and Technology have realized a highly flexible, uniformly luminescent photochromic fiber based on a mass-produced thermal drawing. It not only overcomes the problem of nonuniformity along the transmission direction of traditional waveguiding polymer fibers but also realizes multicolor modulation in a single fiber. This feature meets the demand for highly flexible and mass production of wearable devices and thus is an ideal wearable flexible display. This work shows great potential of the photochromic fiber in wearable human-machine integrations and promotes the field of wearable human-computer interactions.

The photochromic fiber is mainly composed of several key functional materials. It uses a composite material preformed with structural designability to regulate the distribution of its structure within the fiber. This achieves its no-leakage, low-loss waveguiding characteristics, and uniform luminescence in the fiber transmission process. Specifically, the fiber has a light-emitting core made of PMMA (Polymethyl methacrylate) that provides good waveguiding effect. The cladding of the fiber is made of a fluorescent composite material and integrated into the PMMA waveguiding core. The optical waveguide inside the fiber is achieved through the refractive index difference between these two materials, and the pumping of ultraviolet light inside the fiber is converted to visible light through the wavelength conversion effect of the fluorescent composite material (Fig. 1b). This work demonstrates the feasibility of integrating light from pumped fluorescent materials inside the optical fibers, breaking the constraints of traditional photochromic fibers to external light sources. The results provide new insights into light-driven materials and photoexcited material applications, supported by definitive experimental evidence and theoretical calculations.

The fluorescent composite material integrated on the outer side causes a pumping maximum in the visible light distribution power in the transmission direction due to the saturation absorption effect. Additionally, the team was inspired by the RGB three primary colors of the color mixing principle and encapsulated multiple waveguiding cores and different colors of fluorescent materials in a single fiber. By regulating the brightness ratio of the light source in the coupling core, the external emission spectrum of the fiber can be changed. This allows for mapping out the maximum color gamut range that can be achieved by the multicore fiber. Theoretically, tricolor fibers can achieve arbitrary color modulation within this color gamut range. They effectively address the challenges associated with achieving uniform color distribution in the field of luminescent fibers.

To demonstrate potential applications of photochromic fibers as wearable interaction devices, the team designed several scenarios. They designed a wearable wristband that combines sensing and light-emitting interaction functions. It can react with different appearance color effects according to the touch signals and can also be combined with musical beats as a musical ambience accessory. The research team also knitted photochromic fibers of various colors on daily clothes and created an interactive scene for emotion recognition using image acquisition devices and control chips. This demonstrates the feasibility of using the fibers as an auxiliary communication technology tool, ushering in an era of natural, interactive display of information in everyday textiles.

In addition to their excellent flexibility and maneuverability, the photochromic fibers also exhibit impressive robustness and stability. They can achieve highly efficient luminescence not only in strong acid and alkali environments but also in high and low-temperature environments. Additionally, they have excellent washability and friction resistance, making them suitable for most wearable applications. The research results indicate that photochromic fibers are highly flexible with excellent scene universality. It is of great significance for wearable flexible interaction scenes and is expected to bring about a new change in human lifestyle in the fields of communication, navigation, healthcare, wearables, and the Internet of Things.

The development of photochromic fiber technology represents a significant milestone in the fiber industry. This technology uses the thermal drawing method, which is a traditional optical fiber manufacturing process^15^, to mass-produce kilometer-long photochromic fibers. Compared to commercial light-diffusing fibers, photochromic fibers maintain good uniformity in the transmission direction. The pumping light source of photochromic fibers is small, making it easy to integrate into everyday clothing using textile techniques. This work provides new perspectives and inspiration for the field of fiber-based display and interaction, promoting the development of future human-machine integration. The remarkable progress offers a promising path for future exploration and advancement in the emotional interaction and communication field.

## References

[1] Xiong J (2021). Augmented reality and virtual reality displays: emerging technologies and future perspectives. Light Sci. Appl..

[2] Ding YQ (2023). Waveguide-based augmented reality displays: perspectives and challenges. eLight.

[3] Shan QS (2020). Perovskite light-emitting/detecting bifunctional fibres for wearable LiFi communication. Light Sci. Appl..

[4] Kwon S (2018). Weavable and highly efficient organic light-emitting fibers for wearable electronics: a scalable, low-temperature process. Nano Lett..

[5] Tan YJ (2020). A transparent, self-healing and high-*κ* dielectric for low-field-emission stretchable optoelectronics. Nat. Mater..

[6] Shi X (2021). Large-area display textiles integrated with functional systems. Nature.

[7] Larson C (2016). Highly stretchable electroluminescent skin for optical signaling and tactile sensing. Science.

[8] Venema L (2004). A light fabric. Nature.

[9] Selm B (2010). Polymeric optical fiber fabrics for illumination and sensorial applications in textiles. J. Intell. Mater. Syst. Struct..

[10] Bai HD (2020). Stretchable distributed fiber-optic sensors. Science.

[11] Gu FX (2010). Light-emitting polymer single nanofibers *via* waveguiding excitation. ACS Nano.

[12] Di Benedetto F (2008). Patterning of light-emitting conjugated polymer nanofibres. Nat. Nanotechnol..

[13] Zhang ZT (2015). A colour-tunable, weavable fibre-shaped polymer light-emitting electrochemical cell. Nat. Photonics.

[14] Wang LP (2024). Wearable and interactive multicolored photochromic fiber display. Light Sci. Appl..

[15] Rein M (2018). Diode fibres for fabric-based optical communications. Nature.

